# Evaluating Preschool Visual Attentional Selective-Set: Preliminary ERP Modeling and Simulation of Target Enhancement Homology

**DOI:** 10.3390/brainsci10020124

**Published:** 2020-02-22

**Authors:** Amedeo D’Angiulli, Dao Anh Thu Pham, Gerry Leisman, Gary Goldfield

**Affiliations:** 1 Department of Neuroscience, Carleton University, Ottawa, ON K1S 5B6, Canada; 2 Neuroscience of Imagination, Cognition & Emotion Research (NICER) Lab, Carleton University, Ottawa, ON K1S 5B6, Canada; DaoAnhThuPham@cmail.carleton.ca; 3 Department of Systems and Computer Engineering, Carleton University, Ottawa, ON K1S 5B6, Canada; 4 Faculty of Social Welfare and Health Sciences, University of Haifa, Haifa 3498838, Israel; g.leisman@edu.haifa.ac.il; 5 Department of Pediatrics, University of Ottawa, Ottawa, ON K1N 6N5, Canada; ggoldfield@cheo.on.ca; 6 Children’s Hospital of Eastern Ontario, Ottawa, ON K1H 5B2, Canada

**Keywords:** event-related potentials, visual sustained selective attention, voluntary control, self-regulation, executive functions, preschool children, ACT–R, Dipole analysis, spiking simulation

## Abstract

We reanalyzed, modeled and simulated Event-Related Potential (ERP) data from 13 healthy children (Mean age = 5.12, Standard Deviation = 0.75) during a computerized visual sustained target detection task. Extending an ERP-based ACT–R (Adaptive Control of Thought–Rational) neurocognitive modeling approach, we tested whether visual sustained selective-set attention in preschool children involves the enhancement of neural response to targets, and it shows key adult-like features (neurofunctional homology). Blinded automatic peaks analysis was conducted on vincentized binned grand ERP averages. Time-course and distribution of scalp activity were detailed through topographic mapping and paths analysis. Reaction times and accuracy were also measured. Adult Magnetic Resonance Imaging-based mapping using ACT–R dipole source modeling and electric-field spiking simulation provided very good fit with the actual ERP data (*R*^2^ > 0.70). In most electrodes, between 50 and 400 ms, ERPs concurrent with target presentation were enhanced relative to distractor, without manual response confounds. Triangulation of peak analysis, ACT–R modeling and simulation for the entire ERP epochs up to the moment of manual response (~700 ms, on average) suggested converging evidence of distinct but interacting processes of enhancement and planning for response release/inhibition, respectively. The latter involved functions and structures consistent with adult ERP activity which might correspond to a large-scale network, implicating Dorsal and Ventral Attentional Networks, corticostriatal loops, and subcortical hubs connected to prefrontal cortex top-down working memory executive control. Although preliminary, the present approach suggests novel directions for further tests and falsifiable hypotheses on the origins and development of visual selective attention and their ERP correlates.

## 1. Introduction

Relative to goal-directed actions (manifested with behavioral responses), *selective-set* (as defined by Kahneman and Triesman [1]) is a mechanism of selective attention which underlies the ability of detecting task-relevant target information while ignoring (temporally simultaneous or separate) irrelevant information within a sequence of stimuli [1]. In many variations of the selective-set paradigm, speed and accuracy of behavioral response generally improve with age (for review see [2]), and one consistently replicated finding is a sharp developmental transition observed in children between 3 and 5 years of age—he developmental period usually known as “preschool”. This transition is often implied as a critical period for detailing neurofunctional mapping [3,4], and for understanding typical and atypical development of executive attention during the lifespan [5].

The preschool transition has also been reported in studies in which event-related potentials (ERPs) were measured concurrently with different variants of the selective-set paradigm. An important component of selective-set detection is *response inhibition*, which has been studied in the laboratory by using deviant target detection tasks such as the go/no-go task, continuous performance task (CPT) and the stop-signal task [4,5,6]. Specifically, in ERP studies, predominantly in the auditory modality, larger amplitudes of the N2 with (200–300 ms) component have been found for successful responses to no-go trials compared to go trials, which are similar to those found in adults [6,7,8]. However, the N2 component of young children is usually observed between 250 and 500 ms after stimulus onset [9,10] and the no-go N2 effect is larger and more widely distributed across the fronto-parietal electrodes [9,11]. Similar findings in both morphology and latency differences have been reported for other visual processing and visual search tasks [12,13] and in a body of converging findings on error monitoring during visual go/no-go tasks related to error positivity (Pe) and negativity (ERN) components [14,15,16].

To date, Ridderinkhof and van der Stelt [17] conducted the most exhaustive review (including reviews of older studies) of adult and children’s ERP data, which shows that signatures such as the N200 and P300-like (especially the P3b component) were reported in experiments using deviant target detection paradigms such as visual oddball tasks. Importantly, these studies included small convenience samples of 5–6 year-old children (sometimes in groups of broader age ranges, i.e., 4–7 year-olds). Late negative Nc (410 ms) and positive Pc (900 ms) waves (see [18]) as well as Slow Waves were observed in the young participants, and further in conditions where targets were novel stimuli. During the preschool and kindergarten years, the timing of the P300 is significantly slower, peaking on average around 700 ms and ranging between about 600 and 900 ms. Both children and young adults show greater P3 amplitudes to target, attended stimuli relative to non-target, unattended stimuli, and their topographical organizations are qualitatively similar in distribution across posterior electrodes [18].

Based on a modeling analysis of the reviewed studies, Ridderinkhof and Van der Stelt [17] concluded that attentional selective-set is essentially “adult-like” in preschool children, but the age differences in ERP wave morphology and latency may indicate that processing speed and efficiency undergo developmental improvement. Thus, their conclusion implies a form of developmental homology, i.e., an equivalence of structure and functions at two different developmental moments [19]—preschool and adulthood—capturing some neurocognitive aspects of selective-set, but not others (such as, for instance, those related to the execution of response). How this correspondence may practically translate to specific neural mechanisms is still an open question.

According to an influential interpretation [20,21], preschool transition involves a shift from involuntary detection and response towards novelty, to voluntary control of attention and of response to target (including withholding response or attenuating distractors’ interference, or inhibitory control, in favor of more appropriate target response). The shift to voluntary control is usually attributed to the relatively early development of response inhibition and linked with the functional maturation of the frontal system, which is assumed to reflect key changes in the connectivity of the prefrontal cortex (PFC) during the preschool period [22,23,24]. Indeed, the preschool period shows a dramatic maturation of axonal density and myelination of structures supporting visuomotor functions in the frontal-striatum and fronto-basal ganglia networks, and in the fast propagating fibers of the callosal connections of the motor corticospinal system [25,26,27,28,29,30,31,32,33,34].

Voluntary control of visual selective attention has been most recently defined as “top-down” driven (i.e., “regulated by the working memory central executive”) neuronal activity, which is directed to selectively enhance relevant target information and attenuate potential distractors [35]. *Enhancement* is generally associated with larger neuronal and significantly higher electrophysiological activity (see review in [36]) or eye-movement activity [37] concurrent to targets as compared to distractors.

In the present study, we tested the twofold hypothesis that voluntary selective-set detection in preschool children may be associated specifically with enhancement of neural response to target (enhancement hypothesis). Furthermore, we tested the hypothesis that in preschool children this mechanism can be described by some of the similar features, structural and functional, which are attributed to adults (neurofunctional homology).

In the first and second part of the study, we reanalyzed and modeled ERP data from preschool children on a sustained visual detection task, specifically probing set-selection. We expected to find converging evidence that children’s ERP activity concurrent with the target would generally show higher amplitudes, as compared to ERP activity concurrent with the distractor.

In the third part of the study, we extended an ERP-based neurocognitive modeling approach to test the extent to which: 1) The pattern and timing of the preschoolers’ actual ERP responses to target and distractor could be explained by a simulated adult model of ERP activity (functional homology); and 2) the dipoles estimated from preschool children’s ERP activity could approximate the adult spatiotemporal simulation of estimated ERP generators (structural homology).

For periods of the task examined here, the ERP correlates of the selective-set process were confounded with those of the execution of manual response. Nonetheless, by triangulating peak analysis, source dipole modeling, simulation, and spiking modeling, we sought to partition distinct temporal intervals in which we could discriminate with reasonable degree of probability the neural processes recruited predominantly for target enhancement from those recruited for the planning of release/inhibition of response.

## 2. Materials and Methods

### 2.1. Part 1: Reanalysis of Children’s ERP Data

In the first part of this study, we reanalyzed ERP data collected from preschool children who were tested on an adapted computerized version of Akshomoff’s visual sustained detection task [38]. First, using a binning-averaging technique (vincentization), we tested whether ERP amplitudes for targets could be characterized as enhanced activity compared to the ERP amplitudes for distractors. Data vincentization made sure these differences were parametrized; namely, they did not depend on phase or time delays between the two conditions’ waveforms, and neither on other individual’s distribution variations from the grand average distribution.

#### 2.1.1. Participants

Participants were 13 children (9 males, mean age = 5.12, Standard Deviation = 0.75 years) after data additional three children were discarded due to excessive EEG artefacts or inadequate response accuracy level (<75%). Children were recruited as part of an optional follow up to a separate large epidemiological early childhood developmental study [39]. Children were screened with a computerized adapted version of the Peabody Picture Vocabulary Test (PPVT; [40]). Parents completed two standardized age-normed screening assessments: Behavior Rating Inventory of Executive Function^®^–Preschool Version (BRIEF^®^-P; [41]), and the Child Behaviour Checklist for Ages 1½- 5(CBCL/1.5-5; [42]). All children lived in the same neighborhood and came from middle to high socio-economic status families. Participants had normal or corrected to normal vision and had no known, auditory, sensory or cognitive deficits. All participants were typically developing children with no history of medication or referral to disability assessment or services, as ascertained from parental reports and the day care center records. Participants’ scores on all screening measures were within norm.

#### 2.1.2. Visual Sustained Selective-set Attention Task (VSSAT)

**Figure 1:**
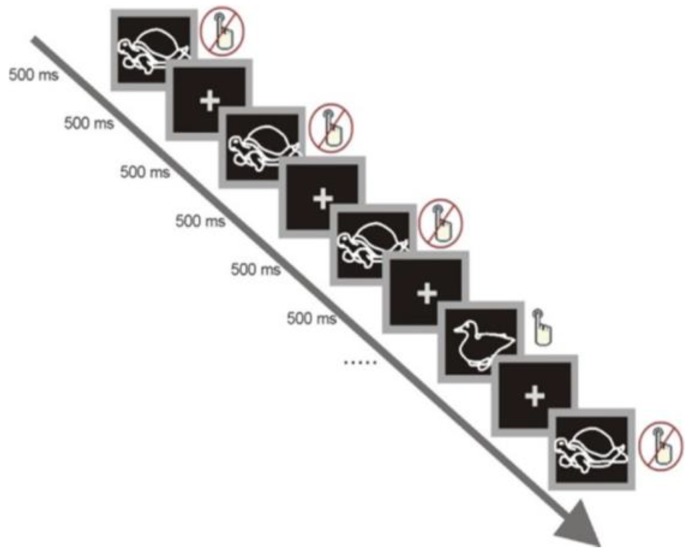
Stimulus presentation and time intervals of each trial in the Visual Sustained Selective-set Attention Task (VSSAT). Adapted from D’Angiulli and Devenyi (2019) [41].

The VSSAT (see Figure 1) was originally selected because: (1) it was validated on samples of similar age and with similar background to the one we previously tested, affording direct comparisons; (2) differently than in most of the typical versions of go/no go tasks, it involves a continuous stream of picture stimuli in short blocks, which allows to determine whether participants are continuously attentive to the target throughout the trial (on correct trials) or when participants cease to attend to the target (on incorrect trials); (3) relative to older subject groups preschoolers can perform it with similar accuracy and engagement levels but show delay in responding to targets, therefore, reaction time measures embedded in this task can be modelled so as to reliably differentiate between the processing stage of visual target selection and the processing stage of the initiation of response to target; (4) this computerized paradigm can be easily used in combination with EEG recording.

**Figure 2:**
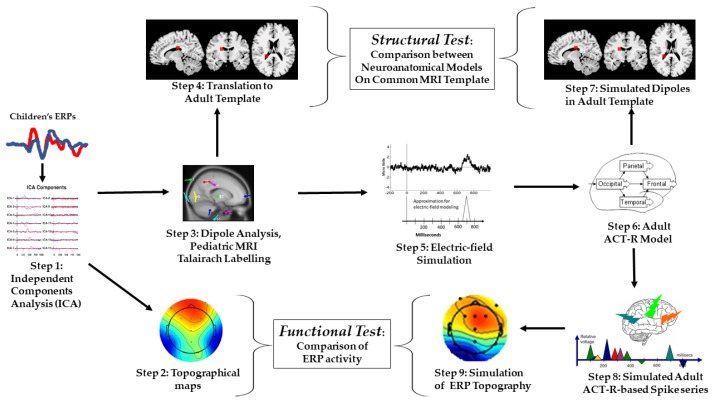
Process flow of the present study. Steps 1–4 describe modeling of actual children’s data; Steps 3–9 describe adult simulations.

Stimuli were presented through the Neuroscan Stim software program (Neuroscan, North Carolina, USA) and displayed on a 19” flat screen monitor. Each trial in the VSSAT consisted of a white outline of a duck or a turtle presented in the center of the monitor on black background and remained on the screen for a duration of 500 ms, followed by a fixation cross for a duration of 500 ms (See Figure 2). Participants were instructed to press a button if the outline of a duck appeared and to refrain from pressing the button if any other image appeared.

#### 2.1.3. Procedure

Upon arrival at the research center, each child’s active assent and signed parental consent was obtained according to protocols approved by the Research Ethics Boards of all participating institutions. Upon parental completion of the in-take assessments, children were tested in a sound-proof electromagnetically shielded EEG booth. Each child was seated at a distance of 58 cm from a 19” flat screen monitor. Children were instructed to respond by pressing a button when they saw a duck and to refrain from pressing the button if they saw any other image.

The entire session consisted of several practice blocks followed by the experimental block. In each block, the duck was shown 25% of the time, while the turtle was shown 75% of the time. Participants proceeded to start the experiment once that they attained 100% accuracy on three consecutive practice blocks. In the experimental block, there were 37 target images and 113 distractor images for a total of 150 trials.

#### 2.1.4. Data Collection and Processing

An elastic cap (Quik Cap, Neuroscan, El Paso, TX, USA) adhering to the standard ten–twenty international system of electrode placement, with recessed Ag–AgCl electrodes (10 mm each) was used for the EEG recordings. The cap montage included 12 electrodes corresponding to electrode reference points at Frontal (F3, Fz, F4), Central (Cz), Temporal (T7, T8), Parietal (P7, Pz, P8), and Occipital (O1, Oz, O2) sites. Horizontal and vertical electro-oculograms (EOG) recorded eye movements with two split bipolar electrodes positioned at the outer canthi for the horizontal EOG and on the suborbital ridge of each eye for the vertical EOG. Previous work and pilot studies in children of similar ages [43,44,45,46,47] suggest no critical loss of reliability in source analysis performed with the present set up.

All impedances were kept below 5 kOhms. Low-pass and high-pass filtering (0.5 to 250 Hz) were applied to the signal prior to digitization. Trials from non-ocular electrode sites that were contaminated by excessive peak-to-peak deflection (i.e., >100 μV or <−100 μV) due to non-stereotypical noise were manually excluded. Brain Electric Source Analysis (BESA v.5.4.28; http://www.besa.de/), an electroencephalographic analysis software package was used to analyze EEG recordings and calculate ERP averages for each of the twelve electrode locations (F3, Fz, F4, Cz, T7, T8, P7, Pz, P8, O1, Oz, O2). Ocular correction was performed using the integrated BESA *adaptive artifact correction* [48] and the *surrogate model* [49]. Principal component analysis decomposition was used to correct for ocular artefacts by selecting components for the horizontal and vertical eye movements as well as eye-blinks. The proportion of rejected trials was less than 15% after artefact correction and removal. ERPs were averaged offline separately for each stimulus type (i.e., target and distractor) at each electrode with all epochs time-locked to the onset of the image and ending at 1000 ms after the onset of the image.

#### 2.1.5. ERP Data Analysis

One known issue in customary peak analysis techniques is that the assumed correspondences are based on subjective selections of time windows which are made post hoc, after examining the data [50]; this issue is exacerbated when young children are compared to adults since analyses often rely on many untested a-priori assumptions on alleged morphological correspondences between peaks of ERPs of young children and the adults. An alternative is to standardize time windows by latency and to examine the entire single ERP waveform across the entire epoch by using automatic blinded analysis procedures such as *waveform binning* [40,51,52,53]. We adopted a hybrid approach in which automatic blinded binning includes traditional peak analysis.

EEGLab [54] was used to analyze EEG recordings and calculate ERP averages for each data point for the twelve electrode locations (F3, Fz, F4, Cz, T7, T8, P7, Pz, P8, O1, Oz, O2). ERPs were averaged offline separately for each stimulus type (i.e., targets and distractors). Each epoch ranged from 200 ms prior to the presentation of the image to 1000 ms preceding the presentation of the image.

EEG sampling rate was 1000 Hz, resulting in a total of 1200 data points for each epoch. Time interval analysis of data points obtained from ERP data were simplified using a standard binning procedure that divided each epoch into bins of equal time intervals. Each epoch was divided into twelve bins of 100 ms time interval. To ensure equivalent data density across bins, the ERP averages across subjects were transformed to vincentized quantile bins [55,56,57]. The computation of the quantile bins was performed assuming the following empirical distribution for the time series of the averaged ERPs:

*F_i_*^−1^(*α*) = *inf*{ −200 < *t* < +1000; *F_i_*(*t*) ≥ *α*}, with 0 < *α* < 1 (1)

The Vincent average of the F_i_s mid-points was then computed as:

∑*w_i_F_i_*^−1^(*α*), where *i* = 1…*n*, and *w_1_* +…+ *w_n_* = 1 (2)

Due to its shape-preservation property, this procedure minimizes the effects of distortions of individual differences in the distributions of averaged ERP peak mid-points, and given that the skewness in our data was modest, it partially offset biases in parametric testing associated with our relatively small sample size (see [57]). Amplitudes sorted within each bin for each epoch were assessed for each of the twelve electrode sites and averaged separately for target and distractor stimuli. To draw a comparison between ERPs for target and distractor stimuli over time, the number of bins for distractors was kept consistent with the target even though distractors did not require a response.

Vincentized amplitudes were analyzed in a mixed model design with interval bins (12 levels) as a between subjects factor and electrode (12 levels) and condition: (2 levels: target vs. distractor) as within subjects factors. To compare differences between target and distractor, focused ANOVA contrasts between mean amplitudes for each electrode and time interval were conducted to obtain the minimum significant standardized absolute difference using the following formula:

Amp diff_µV_*= t*_crit_ (*√*[MSE_within_Σ*(λ_i_^2^/n_j_)*]) (3)

where Abs Amp diff_µV_ indicates the significant difference in amplitude peaks between target and distractor conditions for a given electrode within a given interval bin, *t*_crit_ represents the *t*-value corresponding to the critical *p*-value for determining significance threshold after using a Bonferroni correction for 12 electrodes for multiple comparisons. MSE_within_ represents the error factor for focused *t*-contrasts across all comparisons. This was the within subjects Mean Square error for the interaction between electrode × condition from the omnibus ANOVA. *λ*_i_ represents the contrast weights [58].

#### 2.1.6. ERP Activity Paths

The differences in ERP waveforms between target and distractor conditions can be more easily interpreted via ERP activity path analysis [40,53]. This graphic summary method illustrates the temporal sequence of neural activity between electrode regions while considering differences between target and distractor conditions.

To obtain activity paths, we used the following procedure. All significant average ERP differences for target and distractor conditions observed were compared. The electrode corresponding to the maximum average ERP difference between target and distractor conditions at each interval bin was noted and the brain area corresponding to this electrode was plotted on head maps for target or distractor conditions at each interval bin. If similar (non-significantly different) maximum average ERP differences were observed for two or three electrodes within the same interval bin, areas corresponding to both or all three electrodes were assumed to be simultaneously activated for that interval and were illustrated on the head map.

The principle of *neural wiring minimization* [59] was used as a rationale for plotting the least-path sequence between simultaneous ERP activity electrodes/areas within same interval bin. Accordingly, the following restrictions were applied due to neuroanatomical boundaries: neural activation from occipital electrodes (O1, Oz, O2) could only move forward or laterally, neural activation from frontal electrodes (F3, Fz, F4) could only move backward or laterally. Likewise, lateral activation from left electrodes (F3, T7, P7, O1) could only occur toward the right, whereas lateral activation from right electrodes (F4, T8, P8, O2) could only occur toward the left.

### 2.2. Part 2: Modeling of Children’s ERP Data

In the second part of the study, we run a type of Independent Component Analysis (ICA; [60]) on which we built models of the children’s data. Figure 2 describes the steps in the analysis pipeline. ICA (Step 1) was used to build ERP topographical mappings (Step 2), and then to identify the dipoles corresponding to target and distractor ERPs; this information was then mapped on a pediatric structural MRI template from the Talairach coordinate system (Step 3). Using corresponding neuroanatomical labels and a visual matching procedure, we confirmed and translated the pediatric Talairach coordinates into an adult structural MRI template (Step 4).

#### 2.2.1. Independent Component Analysis (Step 1) and Topographic Mapping (Step 2)

A second Independent Component Analysis (ICA) was performed to identify ERP components using the EEGLab FASTICA algorithm [61]. While the ICA method can estimate location and timings of components, it cannot estimate an absolute magnitude for them since there is an inherent ambiguity between the strength of the component and the attenuation from it to the measurement point. Therefore, the results were converted to topographic maps.

EEGLab includes an editing graphic utility which allows to represent the epochs of averaged ERP onto topographic maps as clips of 10 ms, and then it permits to put these together in sequence resulting in a capture of time course of the dynamic ERP activity. The specific single topographic maps selected for further analysis reflected scalp activity at the mid-points of the time intervals corresponding with vincentized bins corresponding to those described in the ERP activity paths.

#### 2.2.2. Dipole Analysis (Step 3)

The DIPFIT component of EEGLab was used to estimate a set of dipoles in the averaged ERP data that would explain the independent components extracted. Each dipole is assumed to be a region of cortex where several thousand neurons act together in parallel so that their combined electric field is responsible for the EEG signal measured at the scalp. The DIPFIT software usually finds one or sometimes two dipoles for each of the specific regions that appear to produce the independent components.

The EEGLab MRI-based spherical head model with standard age-appropriate (pediatric) Talairach coordinates was selected. The labels of the brain regions which the locations corresponded to were found using the most recently updated version of the Talairach database [62]. We used the built-in function of this software which permits searching for the nearest grey matter within concentric cubes (voxels) from a minimum of ±1 mm up to a maximum range within ±5 mm to exact dipole origin. That is, nearest gray searches involve concentric cube searches with varying diameters. In general, it searches consecutively larger cubes until it finds a gray matter label, with the same outer limit of a 11 mm-wide cube, so it is also possible to find no gray matter labels.

#### 2.2.3. Translation to Adult MRI Template (Step 4)

The pediatric MRI coordinates obtained through the Talairach database [62] were converted to the Yale Bioimage Suite [63] by entering the pediatric coordinates and by matching the anatomical labels. This process was confirmed by visual analysis based on consensus among two independent anonymous judges with expertise in pediatric and adult neuroanatomy.

### 2.3. Part 3: Simulation of Adult ERP Data

In what follows, the descriptions of the components of the analysis pipeline for the third part of the study are organized according to the sequence of steps illustrated in Figure 1. From the information derived from the ICA (Step 1) and the estimated dipoles (Step 3), we derived simple single spiking representations adopting electric-fields estimation modeling (Step 5). Next, the results of Step 5 were used to implement an ERP-based Adaptive Control of Thought—Rational (ACT–R) modeling approach previously validated on the same task [43,44]. We organized the simulated dipoles and the simulated ERP spikes in patterns or chunks of activity corresponding to cortical areas postulated in the adult ACT–R (Step 6). The results of Step 6 allowed us to simulate the sequence of spatiotemporal activity as dipoles mapped onto the same adult structural MRI template as the children’s data (Step 7). This was the basis for comparing children’s and simulated adult estimated localization, therefore, testing for structural homology.

The results of Steps 6 and 7 were also used for aggregate series of simulated spikes to build polyspiking patterns for the simulated dipoles (Step 8). Finally, in Step 9, the simulated data obtained from Step 8 were converted to ERP topographic maps. This final step made possible to contrast children’s and simulated ERP topographic mappings, therefore, testing for functional homology.

#### 2.3.1. Electric-Field Spiking Modeling (Step 5)

To simulate the electrical activity, each module in the neurocognitive model was assumed to be generating one or two dipoles in the location identified in the dipole-fitting stage. The module was assumed to produce its electrical energy in a rising and falling spike. For modeling purposes, a simple triangular wave was assumed, which peaked at the center of the module. The resulting electric field (voltage) was then calculated at the surface of the head for each electrode as the sum of the individual dipole contributions. Elsewhere, we have shown that this method generates reliable, valid and consistent descriptions of actual ERP activity [43,44]. Since the spiking activities of the components occurred at different times in the observed data, it was not necessary to add the effects of more than one dipole at a given time.

**Figure 3:**
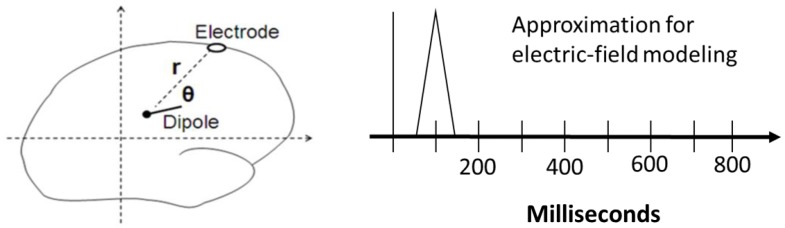
Schematic representation of the calculation of an electric dipole field spiking by simulation; the left panel shows the output of the calculated electric-field potential represented as a simplified spike at fixed timing (determined by the simulated production schedule, here shown at an arbitrary time point for example sake; for the actual simulated timings in the present study see the production schedule shown inTable 1).

The effect of each dipole was estimated in the simulation by following three steps (see Figure 3): (i) The square of the distance r from the dipole to an electrode was calculated by using Pythagoras. Next, (ii) the cosine of the angle θ between the electrode and the dipole was calculated by using vector dot product. Successively, (iii) the electric potential from the dipole at the electrode was derived from Coulomb’s law (=k.p.cos(θ)/*r*^2^), where p is the strength of the dipole and k is a constant. (It was not necessary to know the value of the constant since relative magnitudes were used in the model).

#### 2.3.2. ACT–R Simulation (Step 6)

**Figure 4:**
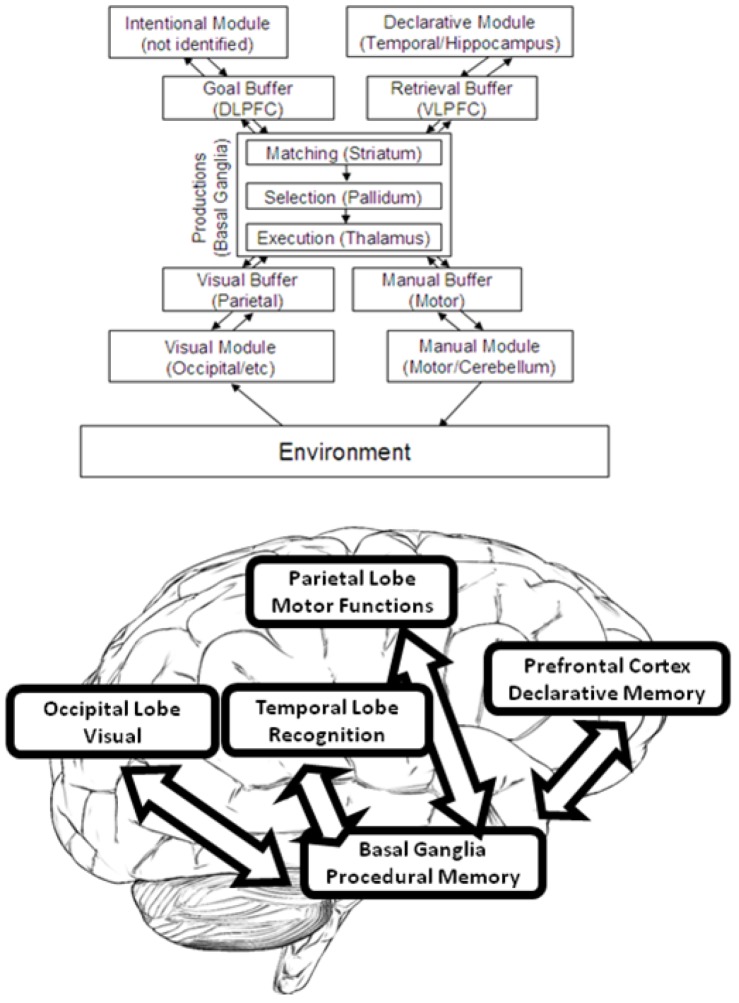
The organization of information in ACT–R (Adaptive Control of Thought–Rational). Adapted with permission from [42].

To model and simulate our data, we used an adapted version of John R. Anderson’s Adaptive (Control of Thought—Rational) ACT–R [64]. In the general architecture, cognition is considered to arise from the parallel interaction of several independent modules. However, top-down processes are directed by the Procedural Module, which is meant to model procedural memory. ACT–R models procedural memory as a production system. Specifically, procedural memory contains production rules (i.e., if/then rules). Communication to and from the Procedural Module is managed by buffers and chunks (see Figure 4). Chunks in ACT–R are lists of predicated information (for example, “duck” could be represented by the chunk: Is-an:animal, Name:duckling, Color:yellow, Size:small).

Each buffer can contain one chunk at a time. Each module has at least one buffer, so there is a visual buffer, a declarative memory buffer, and so on. Modules receive instructions from their buffers and place the results of their activity in their buffers. Collectively, the buffers can be thought of as working memory; they can also be thought of as representing the current context of the task. Productions “fire” when their “*if* condition” matches the contents of the buffers. The “*then*” part of a production then alters the content of the buffers. Productions can only fire one at a time.

In our version of ACT–R, the productions represent electric-field potentials. We programmed the simulation to fit the midpoint of the vincentized bins used to parametrize the ERP data series, so that each production was conditioned to occur at successive steps of approximately t_i_ + 50 ms, with i = 0, 100…, 600. The 50-millisecond cognitive cycle is assumed in many realistic modeling architectures besides ACT–R (for example, Soar, EPIC, GOMS, see [65] for discussion). The neurobiological plausibility of this 50-millisecond cycle has been demonstrated by spiking neural network models simulating well known time constants for the GABA-A receptors in the Basal Ganglia, which in ACT–R (and in various other architectures) is assumed to be responsible for the working memory central executive [66]. Therefore, in the practical implementation of the model we assumed a base production-completion time constant which corresponded to the size of the vincentized bins (100 ms) plus the 50-millisecond cycle constant. Each module contained functions (specifically, ex-gaussian convolutions) to determine activation levels during rest, during the task and the decay rate after firing. Effectively, these functions determined the spiking behavior associated with a given production: Faster productions corresponded to more intense and more quickly decaying spiking.

To implement the simulation, we adopted Python ACT–R [67]. In particular, the present version of the model assumed that the caudate in the basal ganglia acts as the central coordinator (executive) of productions. The hippocampus controls declarative memory while the cingulate cortex controls attention to conflicting stimuli. Frontal cortex supports declarative memory while visual processing takes place in the occipital with further processing in the parietal (see Figure 3). With the time constraints as shown in Table 1, we implemented an ACT–R model which predicted that initially the visual module (occipital) would be activated by the displayed pictures of the target (duck) and turtle (distractor) and would place a representation of the picture in the visual buffer (parietal). Next, the “parietal” representation would be used to retrieve the label of the object and the appropriate instruction about what to do in response to the image of that animal from declarative memory (temporal), which in turn would be placed in the planning buffer (frontal) and initiate the motor program for the manual response, or stop it (basal ganglia).

#### 2.3.3. Adult ACT–R Simulated Dipole Mapping (Step 7)

As in the case of the actual children’s dipoles, the ACT–R simulated dipole data was mapped on a standardized MRI template provided by the Yale Bioimage Suite Web [63].

#### 2.3.4. Adult ACT–R Simulated Spike Series (Polyspiking) (Step 8)

In this step, the simulated electric-field spikes were chunked in ordered series corresponding respectively to the standard localizations and predicted timing of spiking assumed in the programmed schedule for the firing of ACT–R productions (see Table 1). The output of this step was a set of polyspiking patterns including a minimum of six spikes for each of the twelve electrodes. Within each pattern, the spike with maximal activation derived from the dipole of interest at a given bin interval, while all other spikes reflected resting or decaying background activations from the other dipoles. The latter configuration permitted to obtain *coarse coding* which could quantify the amplitude of each spike in the aggregated pattern as a color category (see example in Step 8, Figure 1) corresponding to a standard RGB value so that it could be ordered along an intensity scale.

#### 2.3.5. Adult ACT–R Simulated ERP Topographical Mapping (Step 9)

The simulated topographical maps were obtained through the same utility of EEGLab as the one used for the actual ERP data. To feed the simulated data in a compatible format, the values of the color intensities corresponding to the polyspiking patterns were previously converted (in Stage 8) into an arbitrary relative voltage scale (with range from blue/black, −6 µV, to red/orange, 6 µV).

#### 2.3.6. Actual vs. Simulated Data Comparisons: Homology Tests

##### MRI-mappings and Structural Test

To run the structural comparison, we first estimated the margin of localization error by computing the range differences based on the matches resulting from the search nearest grey area procedure; the measures were in millimeters. Successively, we computed *z*-scores of these ranges (henceforth called *z-Ranges*) which permitted to compare the extent of variations in localization in the actual children’s data against those in the adult simulated estimations.

##### Topographical Mappings and Functional Test

ERP activity comparison between the actual children’s topographic maps and the topographic maps derived from the simulated ERP activity were computed as linear regressions of vincentized averaged ERP amplitudes in the topographic map of children against the corresponding ACT–R simulated data; the fit was assessed by calculating the coefficient of determination, *R*^2^.

## 3. Results

### 3.1. Behavioral Performance

Overall accuracy across all trials was high (Mean percent correct = 89.89%, Standard Error = 1.34%). Mean Hit proportion was 77.5%; mean False Alarm proportion was 6.07% (d’ = 2.31; c = 0.40). Mean response time was 685 ms (SE = 35.64 ms). Age was positively correlated with accuracy (*r(*_13_) = 0.61, *p* < 0.05) but not with response times.

### 3.2. ERP Data

**Figure 5:**
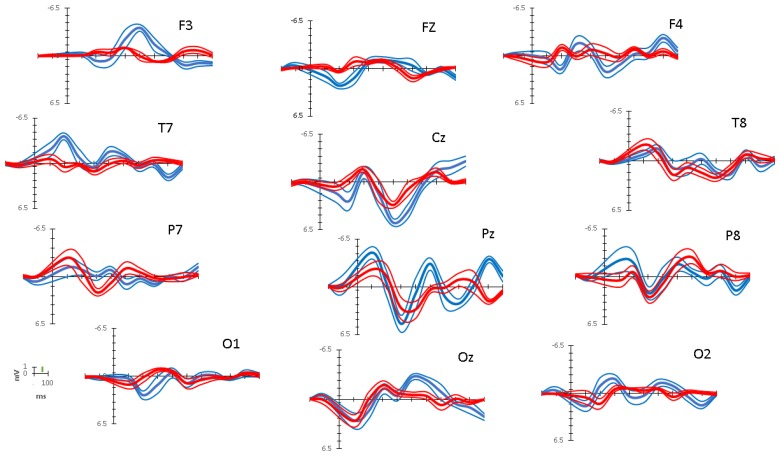
Average vincentized event-related potential (ERP) waveforms for targets (thick blue line) and distractors (thick red line) recorded over twelve electrode locations (F3, Fz, F4, Cz, T7, T8, P7, Pz, P8, O1, Oz, O2) for the epoch ranging from 200 ms prior to onset of each stimulus image to 1000 ms after the stimulus image. Standard errors are represented by thin lines. The microvolt value corresponding to the significance threshold is shown as a green line in scale against the *y*-axis in µV of observed ERP amplitude range at the bottom left legend.

Figure 5 shows overall average vincentized ERP waveforms (thick lines) and standard errors (thin lines) recorded over twelve electrode locations (F3, Fz, F4, Cz, T7, T8, P7, Pz, P8, O1, Oz, O2) from the time interval of 200 ms prior to onset of the image to 1000 ms after presentation of the image for both target (blue lines) and distractor (red lines) conditions.

For all electrodes, within most bin intervals, larger overall ERP peak amplitudes were observed for targets than for distractors. Significant differences in grand peak amplitudes were found (F (1,13068) = 160.124, *p* < 0.01; MSE = 0.468; η^2^ = 0.13), with larger grand amplitudes for targets than distractors. In addition, there was also a three-way interaction between interval bins, electrodes and condition (F (12,13068) = 154.628, *p* < 0.01; MSE = 20.367; η^2^ = 0.13).

Given the three-way interaction, to compare differences between target and distractor conditions across electrode locations and time intervals we then performed Focused ANOVA *t*-contrasts (see Section 2.1.5). The t-value corresponding to the critical p-value for determining significance threshold after using a Bonferroni correction was t_crit_ (12) = 5.69 ((MSE = 0.132); *p* = 0.0001 (two-tailed); η^2^ = 0.71). Accordingly, the minimum significant amplitude difference between standard and distractor peak amplitudes was computed to be 0.80 μV. The significance threshold is shown in scale against the *y*-axis µV legend in Figure 5.

We also performed traditional peak analysis, which is presented in the Supplementary Materials. There were no substantial discrepancies between the two analyses.

Table 2 reports the differences of average peak ERP amplitudes in target and distractor conditions (peak amplitude target − peak amplitude distractor) within interval bins of 100 ms for all electrodes. This analysis focused only on bins capturing processes before motor responses, that is, up to the approximate time of the occurrence of manual response for most children (bins including data up to 700 ms) to exclude effects that might be attributed to motor responses (i.e., when participants responded to targets).

Inspection of Table 2 confirms that the effects in most of the significant pairwise comparisons (46 out of 84) involved higher amplitudes for targets as compared to distractors. In contrast, larger amplitudes for distractors over targets occurred only for much fewer comparisons (5 out of 84). The difference between the proportions of significant target enhancement (55%) vs. distractor (6%) is substantial (χ^2^(1) = 45.05; *p* < 0.0001).

### 3.3. ERP Activity Paths

**Figure 6:**
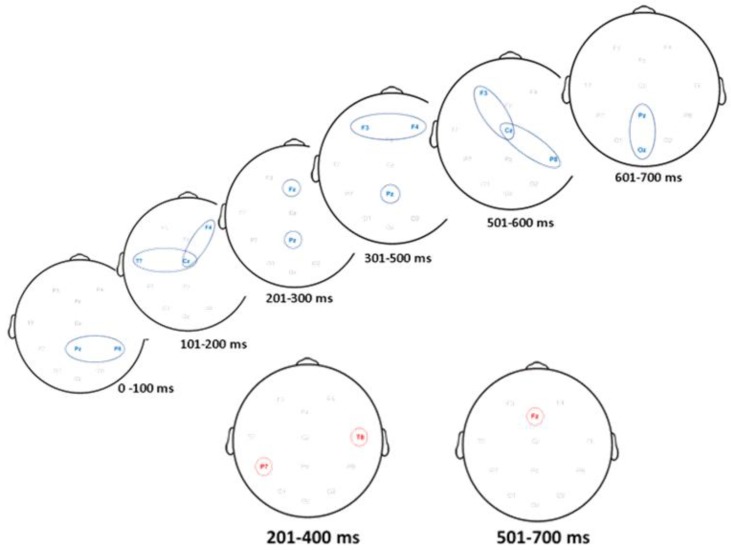
Neural paths of maximum ERP activity over seven interval bins of 100 ms time intervals. Top Panel: Target. Bottom Panel: Distractor.

The results of the ERP activity paths analysis are shown in Figure 6. Neural paths of maximum mean difference in neural activity for targets are shown in blue and neural paths of maximum mean difference in neural activity for distractors are shown in red. The paths were constructed from the results of Table 2.

With reference to the onset of target, ERP paths based on maximum mean differences in peak amplitudes first occurred at the parietal central and right electrodes and moved anteriorly to the left temporal, central and right frontal electrodes. Activation subsequently occurred at the frontal and mid parietal electrodes and finally moved to central parietal and occipital electrodes. For the distractor, ERP pathways based on maximum differences in peak amplitudes were only detected at the left parietal and right temporal electrodes. Neural activation finally moved to the mid-frontal electrode.

### 3.4. Comparison of ERP Activity and Localization: Preschool Data vs. Adult Simulation

**Figure 7:**
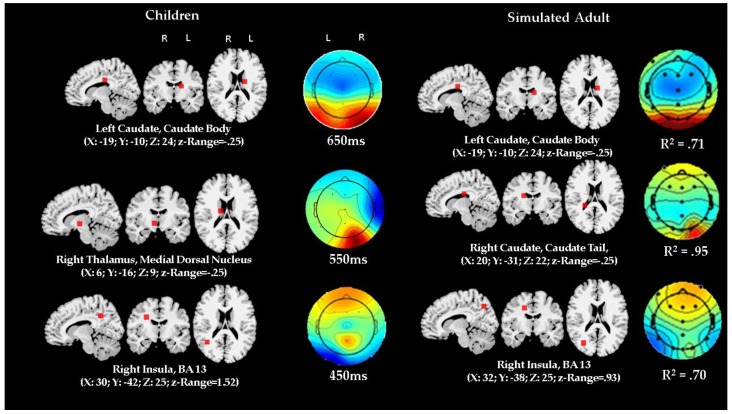

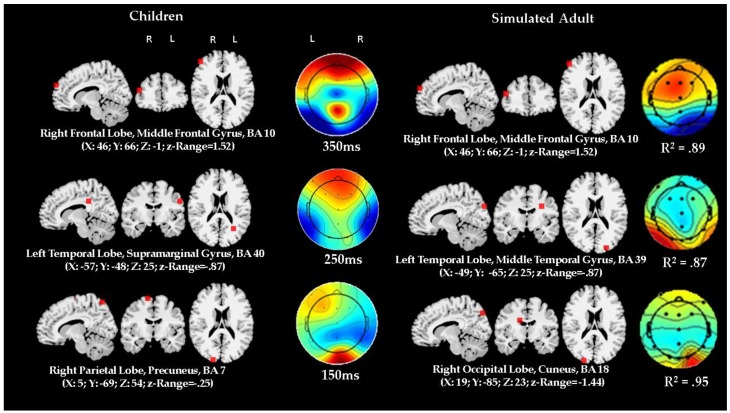
Comparison between dipole source analysis and topographical mappings of actual preschool children ERP data and simulated ERP data by using an ACT–R modeling architecture in the target condition. Timings are set by the ACT–R model module production schedule simulation (given in Figure 3). Coefficient of determinations show fit results for actual and simulated topographic maps comparisons. “R” represents right, and “L” represents left (Please note that lateral side of brain is showed opposite to perspective of the observer in MRI scans). Z-range represents range of normalized Talairach coordinates and is a measure of margin of error expressed as z score; comparisons between the z-ranges in actual and simulated data showed no significant differences.

**Figure 8:**
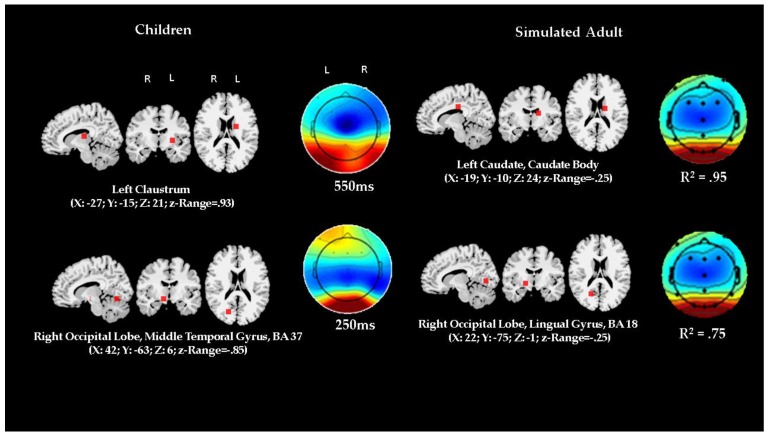
Comparison between dipole source analysis and topographical mappings of actual preschool children ERP data and simulated ERP data by using an ACT–R modeling architecture in the distractor condition. Timings are set by the ACT–R model module production schedule simulation (given in Figure 3). Coefficient of determinations show fit results for actual and simulated topographic maps comparisons. “R” represents right, and “L” represents left (Please note that lateral side of brain is showed opposite to perspective of the observer in MRI scans). *Z*-range represents range of normalized Talairach coordinates and is a measure of margin of error expressed as *z* score; comparisons between the z-ranges in actual and simulated data showed no significant differences.

Figure 7 and Figure 8 report the comparison between the localization of dipoles for distractor and target trials. The figures also show the ERP topographical maps of the actual children’s data contrasted with the adult simulation data.

The *z*-scores of the variation in matched anatomical localizations did not show significant differences between the children’s and the adult simulated data. Similarly, there was a strong fit between the topographic maps of the actual observed children’s data and the simulated adult data. For both target and distractor condition, in the regressed data fitting model between actual and simulated data, the coefficients of determination ranged similarly from *R*^2^ = 0.70 (F (1,11) = 23.65; *p* = 0.0005) to *R*^2^ = 0.95 (F (1,11) = 148.30; *p* < 0.0001), indicating very strong correspondence.

Overall, the results show that the ACT–R model has a very good fit with the actual ERP data, however, two discrepancies are of note. The MRI Talairach coordinates did not match in one instance out of six comparisons concerning the target data, although the spatial coordinate variation was moderate and within satisfactory margins. The match was more imprecise for the distractor data where in both comparisons the Talairach coordinates referred to very proximal but still distinctly different anatomical structures. In addition, differences between ERP time latencies in the actual data and the one derived by simulation might have worsened the fit statistics, since the time of ERP occurrence predicted by ACT–R might actually have not led to sample the most optimal actual data to be fed to the simulation algorithm.

## 4. Discussion

In the present study, preschool children’s performance on VSSAT replicated previous behavioral results [38]; their concurrent ERPs showed that, for most electrodes, the amplitudes were more pronounced in target than distractor trials from about 50 ms to 650 ms post-stimulus presentation. However, response to distractor had relatively higher amplitude than targets in right temporal and left parietal electrodes in the interval ranges of 200–400 ms and in the midfrontal electrode at 500–700 ms. These exceptions concerned a narrow subset of electrodes, for a narrow time range, with relatively smaller effects, and might be interpreted as correlates of *interference control*, namely, resistance to interference from the distractor and suppression of its impact on ongoing selective-set processing aimed at releasing or inhibiting the appropriate (correct) manual response [68].

In the task we used, the ERP correlates of enhancement were confounded with those of the execution of manual response only after 400 ms. The determination of response times within ±2 standard deviations from the grand mean (685 ms) reveals that manual response in most cases could be estimated to occur between 425 and 945 ms. Therefore, selective-set effects were unconfounded by manual motor processing across bin-intervals up to 400 ms. Within this time interval, the peak analysis showed evidence of enhancement of ERP to target except for the two instances mentioned. This analysis also corroborated older findings in showing anteriorly and centrally distributed adult-like N200 and N400 and posteriorly distributed P100 and P300 waveforms. Therefore, we conclude that the present findings support a slightly amended version of the enhancement hypothesis in that a parallel relatively minor and segregated processing may have occurred in a subnetwork in order to suppress the distractor’s interference.

By triangulating response times analysis, peak analysis, source dipole modeling, simulation, and spiking modeling, we provided converging evidence separating the selective-set processes as occurring earlier (before 400 ms) than the actual response (at around 700 ms, on average) and showing yet another distinct spatiotemporal pattern of activity from those associated with later processes (between 500 and 650 ms), which presumably reflected the planning and preparation of response.

We also performed activity paths analysis to illustrate plausible sequence of the prominent neural activity over time for target and distractor based on maximum differences in peak amplitudes. The results seem compatible with the interpretation that attending the target while holding in working memory the response plan yielded a neural path starting from the parietal regions, then to right temporal and central regions and finally to the frontal regions. In contrast, withholding responses to distractor seemed associated with punctuated activity at the left parietal and right temporal regions. Importantly, comparison of neural activity between target and distractor reveals that in the initial 400 ms there was less involvement of frontal areas concurrent to the distractor.

Dipole analysis further suggests possible and plausible neurofunctional pathways and dynamics involved at cortical and subcortical level. This analysis (see Left Panels of Figure 7 and Figure 8) estimated that initially, within the first 150 ms, the generators of scalp ERP signals, which are distributed posteriorly at right and mid parietal electrodes, seem to correspond to activity in the precuneus. Subsequently, around 250 ms, the ERP activity, mapped on the scalp at left temporal, central, and right frontal electrodes, seems to correspond to a dipole source in the left supramarginal gyrus of the left temporal cortex. Before what might be reasonably considered the timeframe for implementing the manual response, around 350 ms, ERP activity mapped at mid parietal and frontal electrodes was estimated as being generated at the level of a dipole located in middle frontal gyrus of the right frontal lobe. At approximately 45 ms, ERP activity was then estimated to a source in the right insula. The following ERP activity, at 550 and 650 ms, was associated with dipoles in the medial dorsal nucleus of the right thalamus and the right caudate body, respectively.

In contrast, ERP activity concurrent to the distractor was accounted for by just two dipoles, the first occurring at 250 ms in a source located in the middle temporal gyrus, at the junction of right occipital and temporal cortices, the second occurring at 550 ms in the left claustrum.

The previous analysis converges with the findings of a close fit between the preschool children’s ERP topographic as well as derived source data and the adult-based ACT–R functional and structural simulations. This convergence suggests evidence of neurofunctional homology. In addition to providing preliminary validity and reliability to the analysis based on the actual data, the converging results from the ACT–R modeling may provide a framework for interpreting the results in a coherent meaningful way and for a detailed inferential reconstruction of the plausible, possible underlying neurocognitive processes related to the enhancement mechanism.

As modeled through ACT–R, our findings may be interpreted as showing that in the initial phase of the VSSAT, target presentation might have been associated with the involvement of key structures of the dorsal (precuneus, BA 7) and ventral (supramarginal gyrus, BA 40) attentional networks [69,70,71]. These structures appeared to be activated relatively early, similarly than in adults. Next, activation seemed to follow in two functionally interconnected parts, one in the dorsolateral prefrontal cortex (middle frontal gyrus, BA 10); the other in the frontal part of the dorsal attentional network (insula, BA 13). The literature indicates that these structures seem to be activated during the processing of deviants and standards, and specifically, both structures seem to be involved in voluntary target detection, playing important roles in top-down selective attentional control [72,73,74]. Successively, activation seems to have involved the thalamic dorsomedial nucleus, which might play a role in the regulation of cortical networks, especially when the maintenance and temporal extension of persistent activity patterns in the frontal lobe areas are required, as in the case of sustained attention [75]. The final stage leading to manual response seemed to be associated with the involvement of a key structure in basal ganglia-striatum network, the caudate body. This “cognitive” part of the caudate seems to participate in the control of action including executive functions such as working memory, set shifting, and inhibitory control [76,77,78].

The presentation of the distractor seemed to be associated with early engagement of the middle temporal gyrus (BA 37) which is generally deemed to be involved in visual recognition and verbal labeling/categorization [79]. Subsequent late activation seemed to involve the claustrum, a structure thought to participate in the regulation of vigilance and in voluntary allocation of attention [80].

Triangulation of peak analysis, ACT–R modeling and simulation for the entire ERP epochs up to the moment of manual response (~700 ms, on average) suggested converging evidence of distinct but separate interacting processes of enhancement and planning for response release/inhibition, respectively. Thus, the results from the triangulation, considering both target and distractor conditions, overall suggest potentially important interrelations between basal–parietal–temporal–frontal–basal loops and large-scale attentional networks. The feedback loops and functional connectivity originating and ending in the basal ganglia and the striatum, as postulated by the ACT–R architecture, control diverse behaviors largely but not exclusively involved in high-level perception, walking, talking, thinking, language, comprehension, associated with frontal lobes, where the motor strip also sits [81]. As some theories have proposed [82], the aspects which might undergo fine-tuning in young children, in terms of speed and efficiency, most likely may not involve the selective-set in isolation, but rather its voluntary and flexible coordination with high-level perceptual and working memory processes, recruited for selecting, monitoring and executing or inhibiting the appropriate behavioral response.

A brief discussion of the main limitations and caveats of our study is in order. While the number of electrodes and montage set-up we used may be justified for dipole source analysis in young children (as we have detailed in the methods section and especially for practical challenges in collecting EEGs in this population), it may not be sufficient for reliable source analysis in adults. Therefore, the results need to be replicated in further studies which combine real fMRI and possibly eye movement measurements. Furthermore, as we have already noted ACT–R simulation could be refined (or even replaced by a more flexible architecture) to improve fit and predictions in timings and anatomical localization of neurofunctional modules.

Lastly but not least, although our modeling and simulation procedures are grounded in the literature, being validated by separate tests of the most relevant components of ERPs from actual adult samples [83,84,85], still we did not report data from an actual adult comparison group. While we acknowledge that in this respect our results are preliminary, and indeed this is a desirable priority for future research, adult comparison on this version of the VSSAT might not necessarily augment the strength of the supporting evidence because it presents non-trivial methodological challenges. In particular, we have learned from small pilot studies in our lab that the current VSSAT is not appropriate for adults. This task needs to be adapted to prevent confounds of other spurious aspects (i.e., boredom, engagement level, ceiling effects) occurring in adult participants (but not in preschool children). In other words, this task should be modified significantly to “equate” children’s state and age-appropriate task demands. However, as pointed out by others (notably, see [17]), if children and adults are not compared on the same task, data interpretation would still depend on assumptions derived from a priori hypothetical models. Consequently, under such differing conditions, the correspondence defining homology on the basis of comparisons between actual adult and children ERP data would still be based on a type of model-mediated inductive inference. This would be essentially similar to the present approach, therefore, the resulting evidence would not be more “direct” or “realistic” or logically different than the one offered here.

## 5. Conclusions

In summary, the pattern of results invites the conclusion that preschool children’s ERPs associated with visual attentional selective-set were enhanced in response to target as compared to distractor, and may have involved functions and structures consistent with adult ERP activity. The modeling results suggest a large-scale network, including Dorsal and Ventral Attentional Networks, corticostriatal loops, and subcortical hubs connected to prefrontal cortex top-down (working memory) executive control. The present findings are compatible with the claim [17] that the attentional selective-set might be, or become, adult-like by 4–5 years of age. Although preliminary, our approach may contribute to suggest novel directions for further tests and falsifiable hypotheses on the origins and development of visual selective attention and their ERP correlates.

## Figures and Tables

**Table 1:** Simulated locations and predicted timing of spiking occurrence according to the present adapted Python ACT–R architecture.

Function	Brain Region	Time (ms)
Visual processing	Occipital	150
Spatial attention	Parietal	250
Declarative	Temporal	350
Executive	Frontal	450
Procedural	Basal ganglia	550
Manual	Parietal	650

Note: The timings shown in the table include the base constant and the increments from the 50-millisecond cognitive cycle (described in Section 2.3.2) and each of them represent the approximate estimated moment in which a given production process in the ACT–R model is completed.

**Table 2:** Differences of average peak ERP amplitudes and corresponding latencies in target and distractor conditions (absolute value of (peak amplitude target – peak amplitude distractor)) within interval bins of 100 ms of epoch range (0 to 700 ms) for all electrodes.

**Interval Bins**							
	(0–100 ms)	(101–200 ms)	(201–300 ms)	(301–400 ms)	(401–500 ms)	(501–600 ms)	(601–700 ms)
**Mean Differences in Peak Amplitudes (Target – Distractor) in μV**							
**Frontal Network**							
Left (F3)	−0.142	1.280 ^T^	0.809 ^T^	−1.198 ^T^	−3.855 ^T^	−2.266 ^T^	−0.686
Midline (Fz)	1.298 ^T^	1.997 ^T^	2.275 ^T^	−0.101	−0.109	−0.876 ^D^	−1.781 ^D^
Right (F4)	−0.550	2.962 ^T^	−1.617 ^T^	−0.024	2.493 ^T^	1.017 ^T^	0.800 ^T^
**Centro-Temporal Network**							
Left (T7)	−1.644 ^T^	−4.249 ^T^	0.095	−1.375 ^T^	−0.568	1.067 ^T^	−0.782
Midline (Cz)	0.805 ^T^	2.976 ^T^	0.436	0.371	2.356 ^T^	3.061 ^T^	1.074 ^T^
Right (T8)	1.240 ^T^	−0.646	−0.946 ^D^	−0.148	−1.806 ^T^	−0.749	0.753
**Parietal Network**							
Left (P7)	1.366 ^T^	−0.106	−2.172 ^D^	−1.772 ^D^	2.118 ^T^	0.870 ^T^	0.822 ^T^
Midline (Pz)	−2.256 ^T^	0.993 ^T^	2.139 ^T^	−2.181 ^T^	−3.538 ^T^	1.241 ^T^	3.268 ^T^
Right (P8)	−2.720 ^T^	−1.200 ^T^	−0.526	−0.620	0.293	2.100 ^T^	0.846 ^T^
**Occipital Network**							
Left (O1)	−0.882 ^T^	2.402 ^T^	2.020 ^T^	0.147	−0.059	−0.677	−0.314
Midline (Oz)	−0.045	0.060	0.561	0.388	−2.287 ^T^	−2.029 ^T^	−1.690 ^T^
Right (O2)	1.196 ^T^	−2.431 ^T^	−1.358 ^T^	1.062 ^T^	0.729	−0.742	−1.466 ^T^

Note: Superscripts indicate significant differences in peak amplitude between target and distractor condition: “T” indicates larger amplitude for target; “D” indicates larger amplitude for distractor.

## Supplementary Materials

The following are available online at https://www.mdpi.com/2076-3425/10/2/124/s1.
