# Supplementary figures and images for: Evaluating Preschool Visual Attentional Selective-Set: Preliminary ERP Modeling and Simulation of Target Enhancement Homology
Source: Brain Sci. 2020 Feb 22;10(2):124. doi: 10.3390/brainsci10020124 (PMC7071495; doi:10.3390/brainsci10020124)

# F3

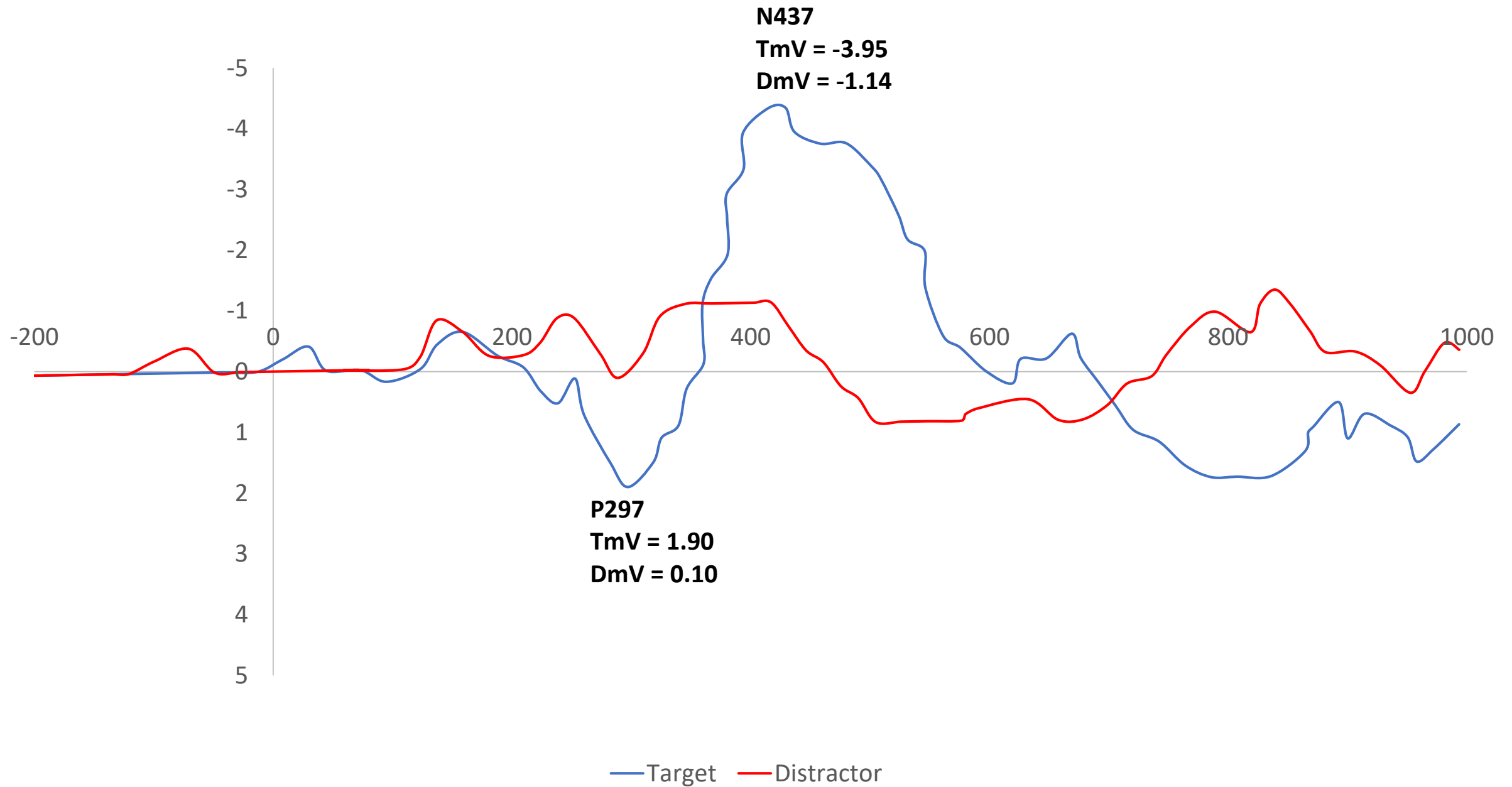

# FZ

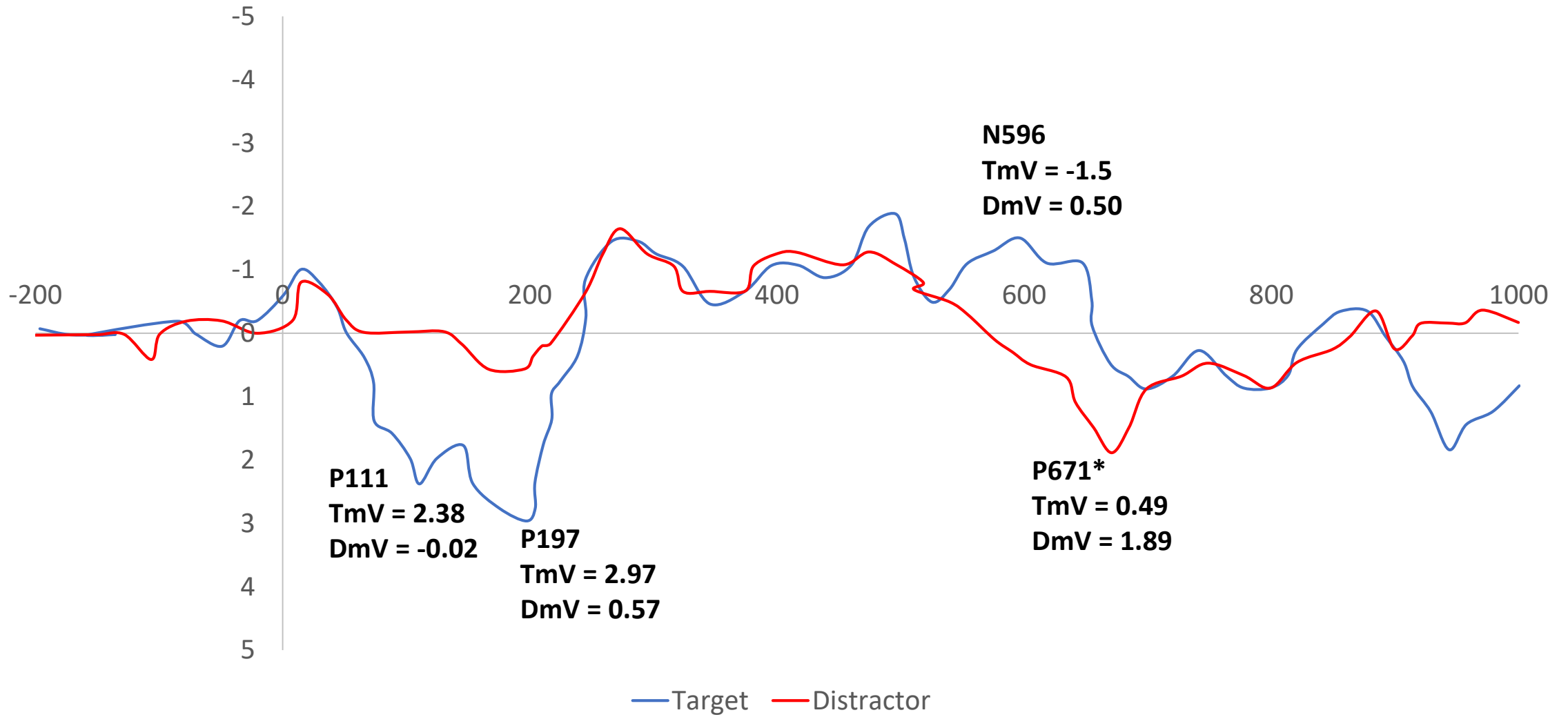

# F4

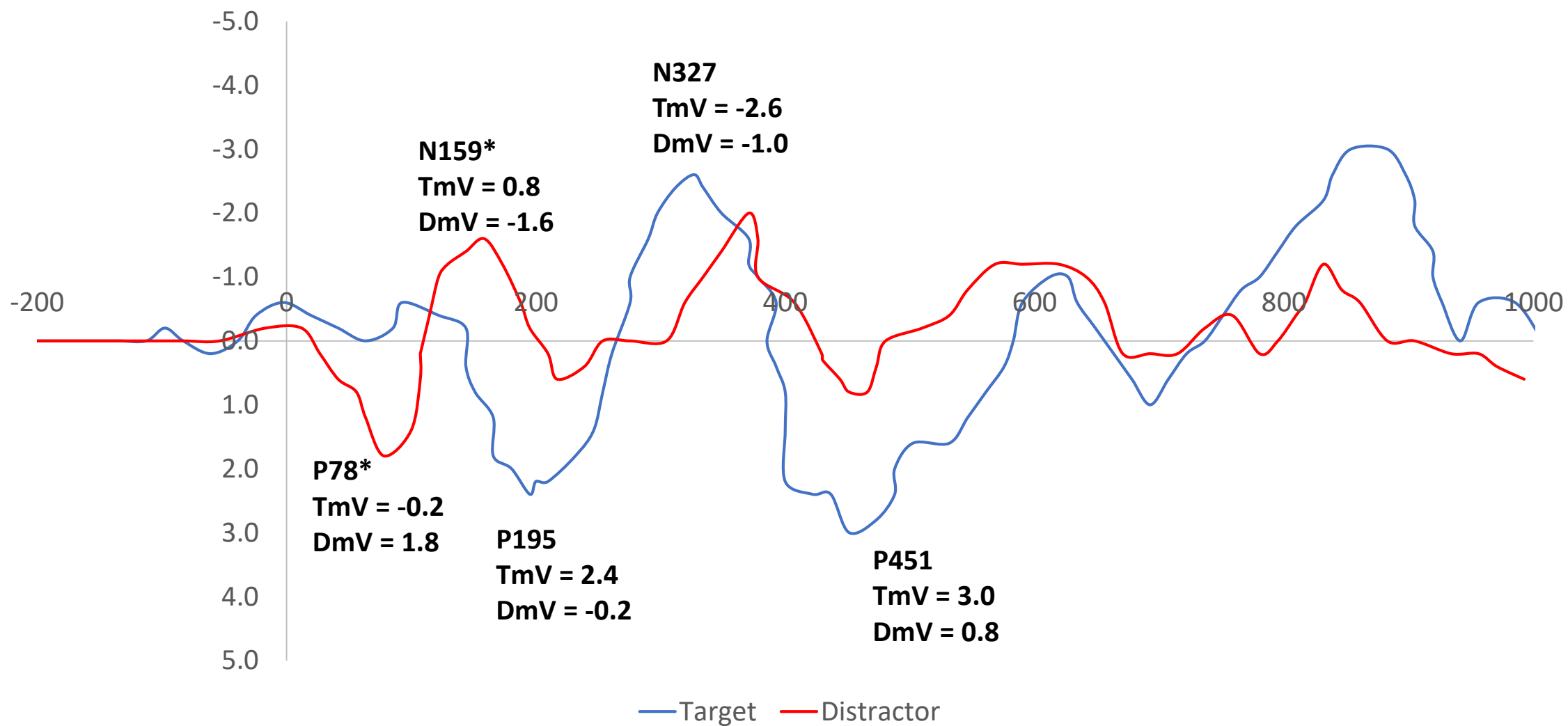

T7

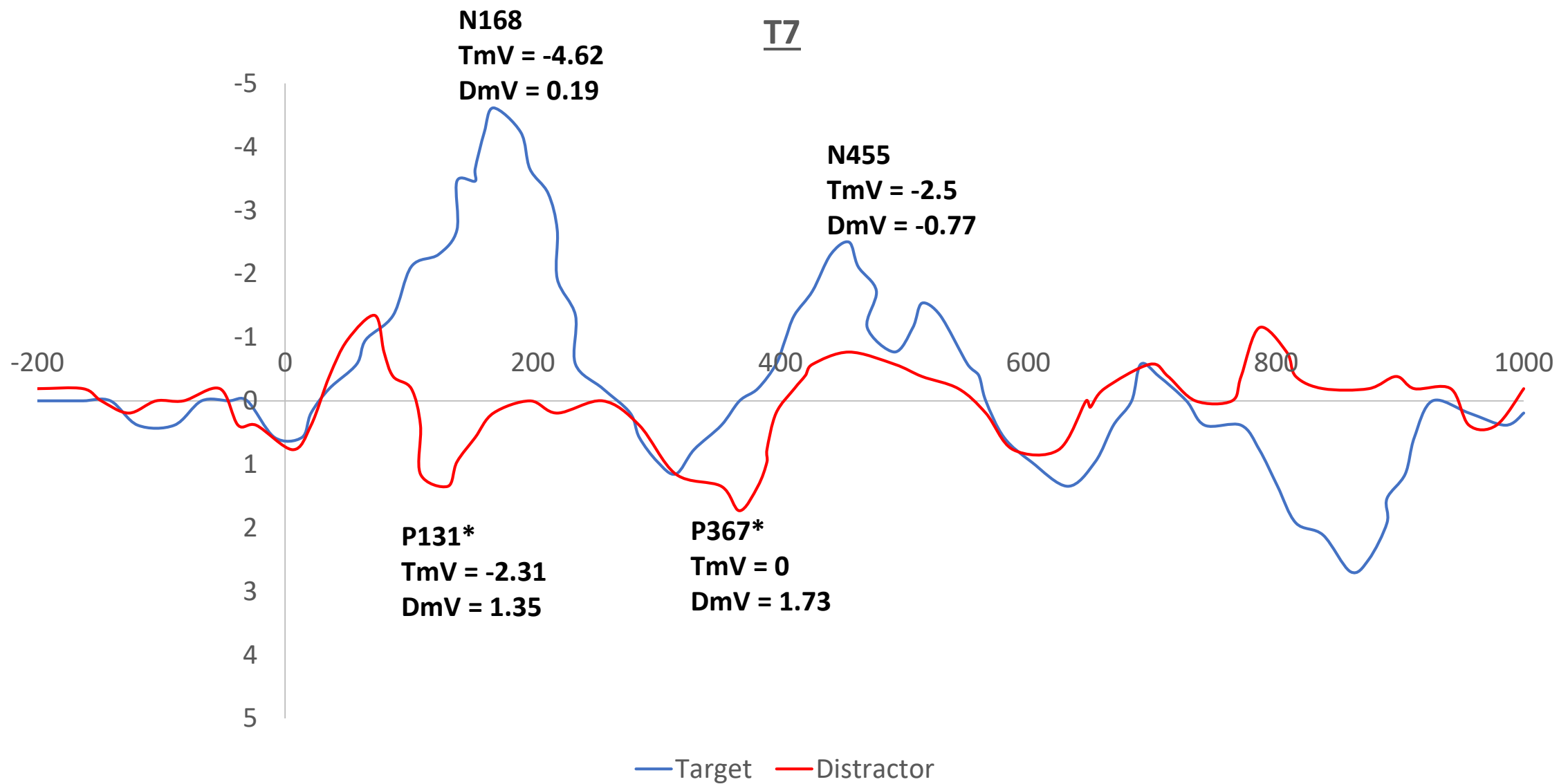

CZ

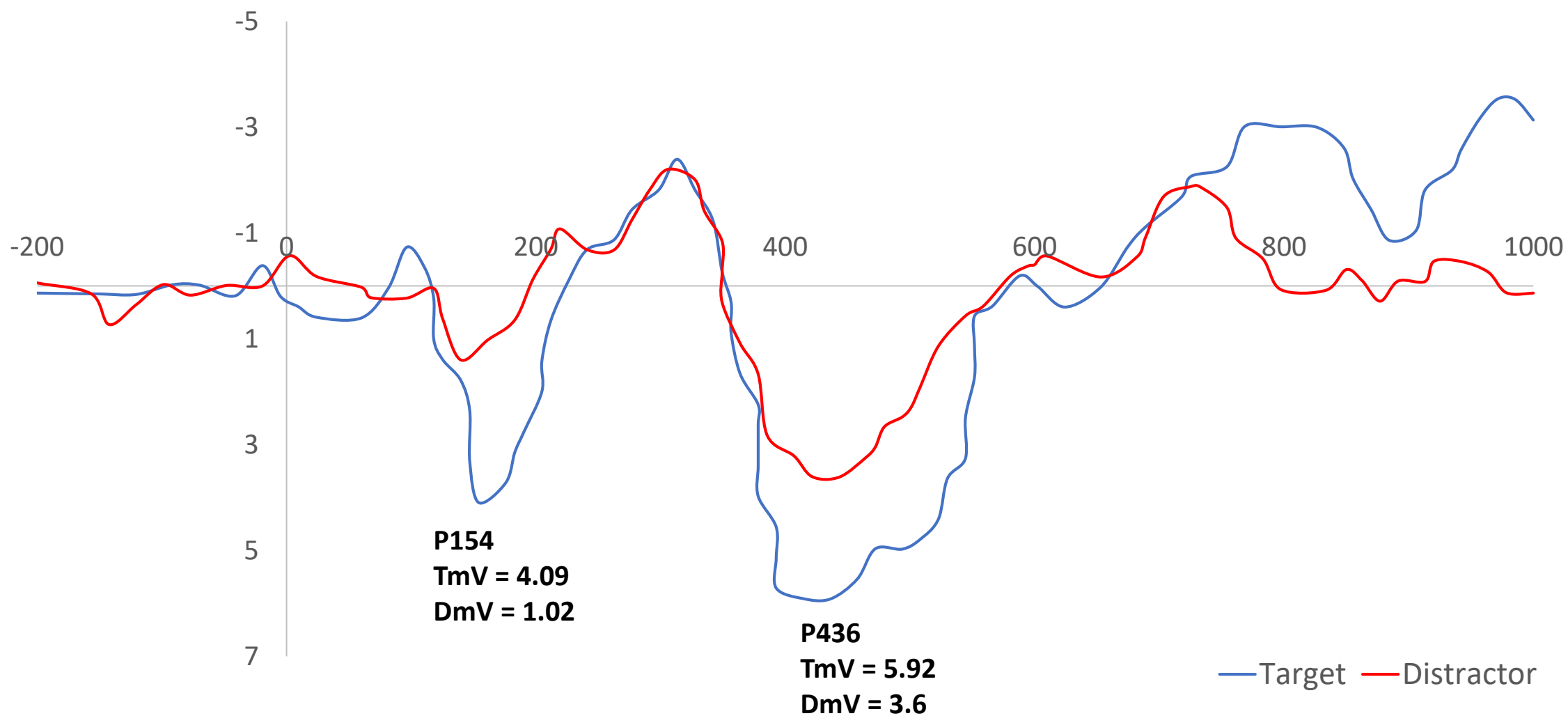

## T8

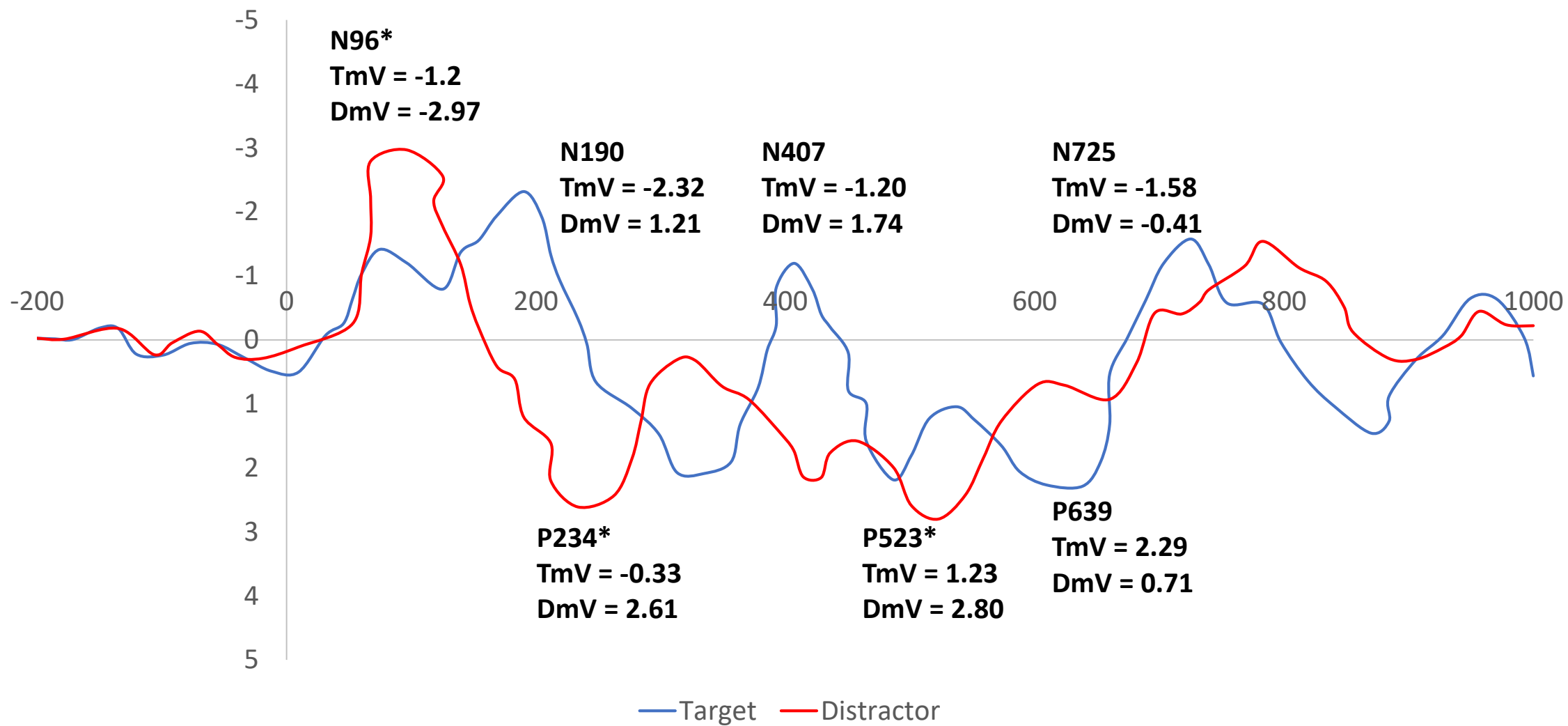

P7

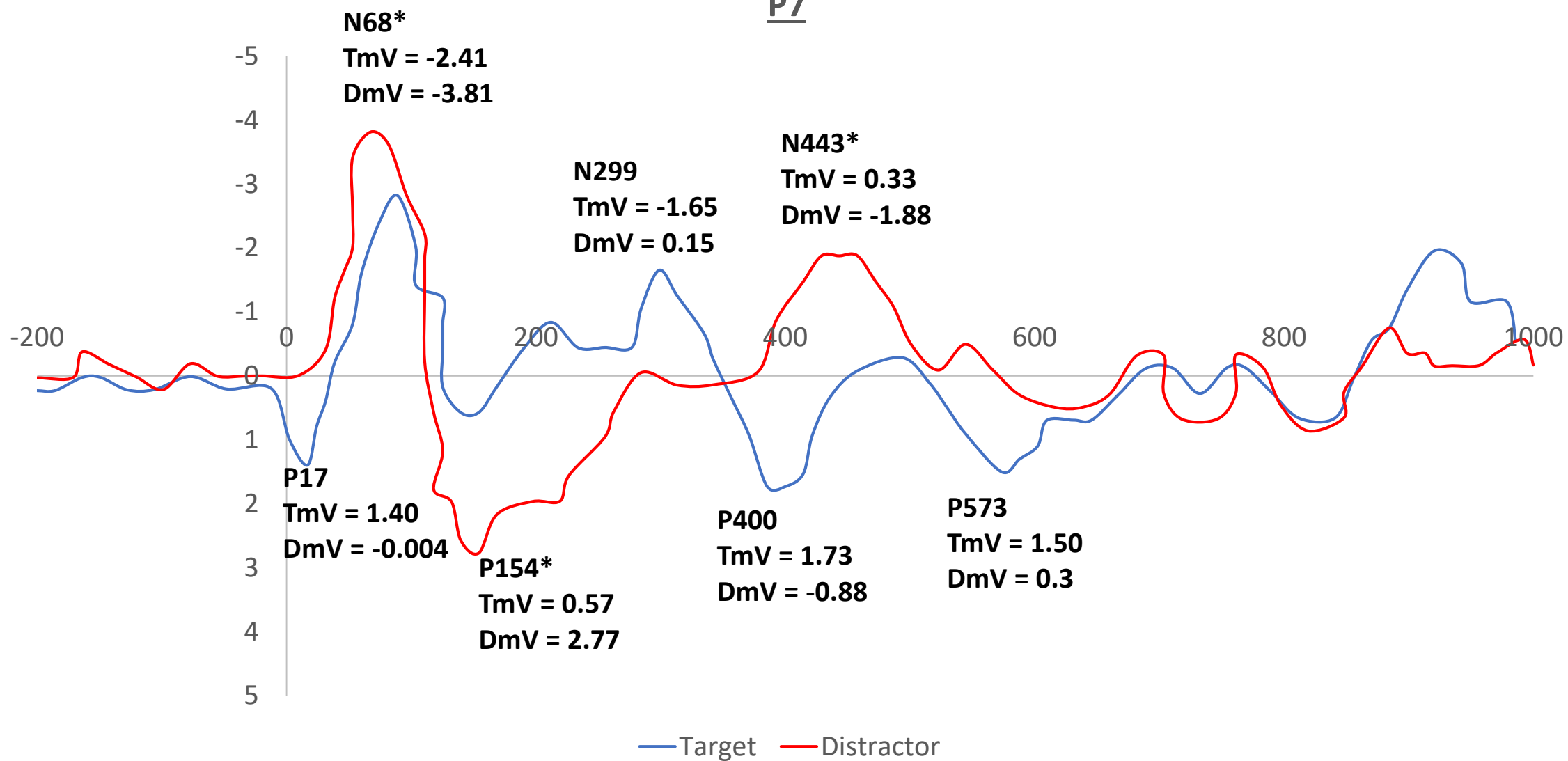

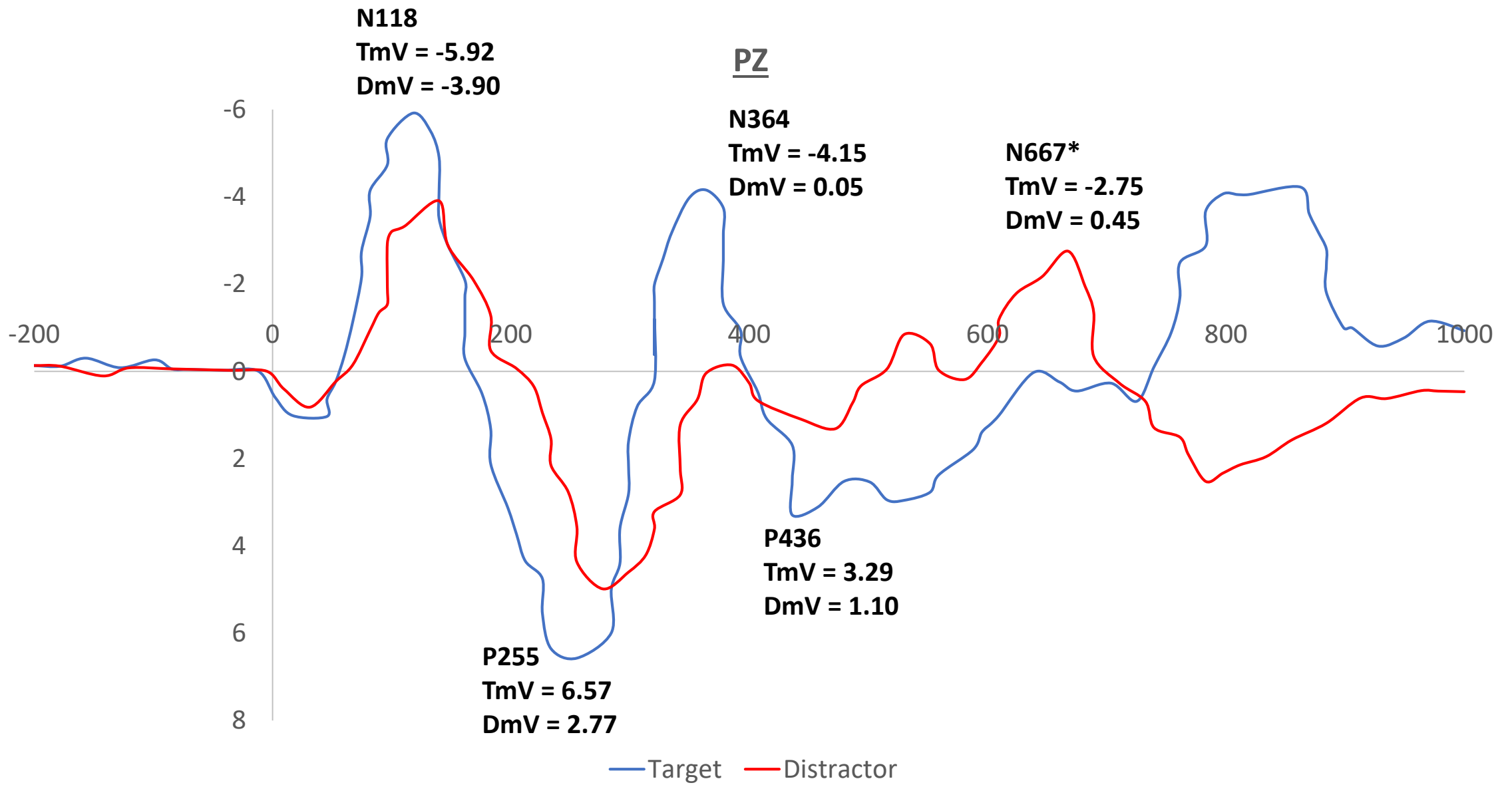

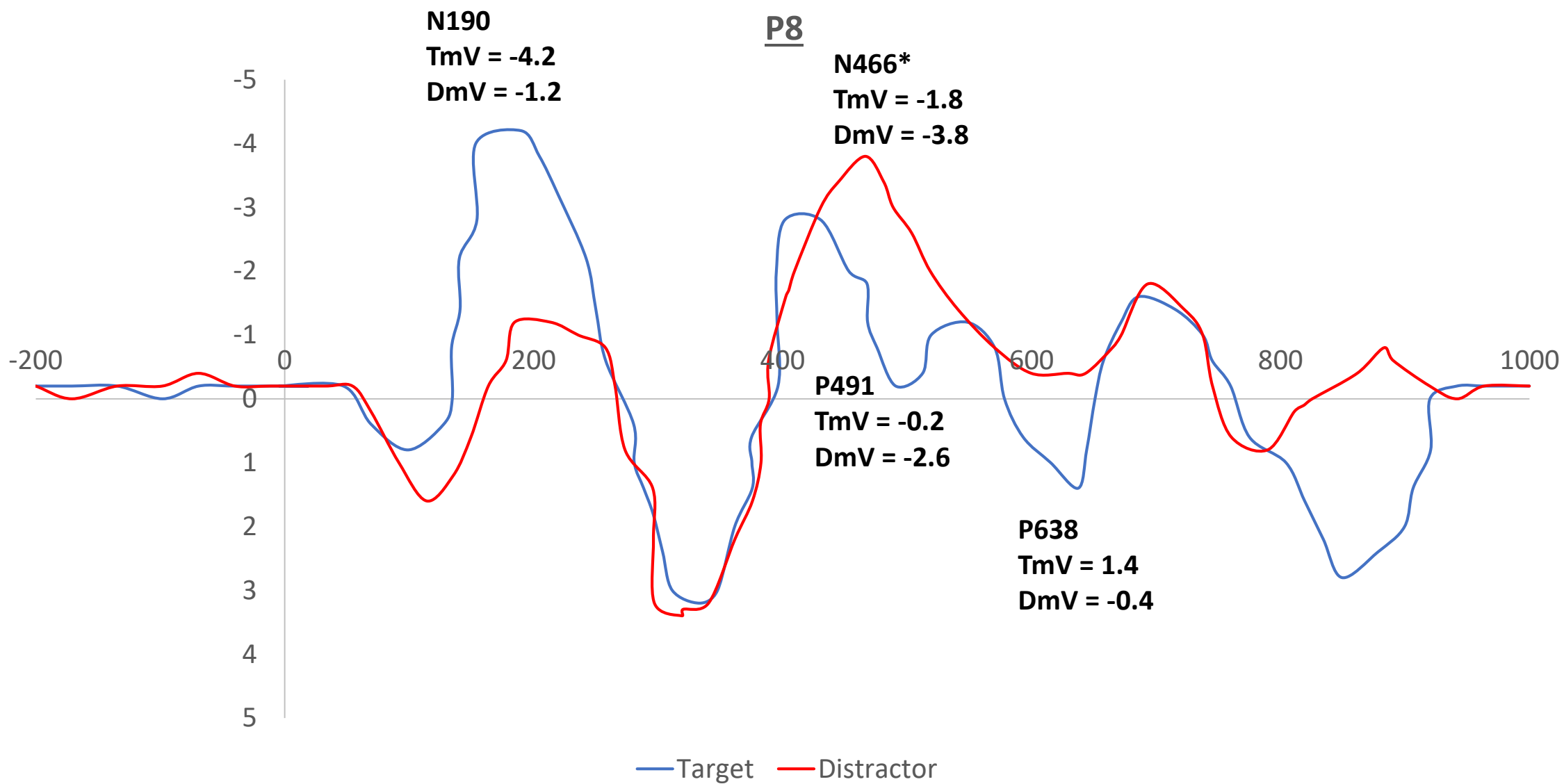

O1

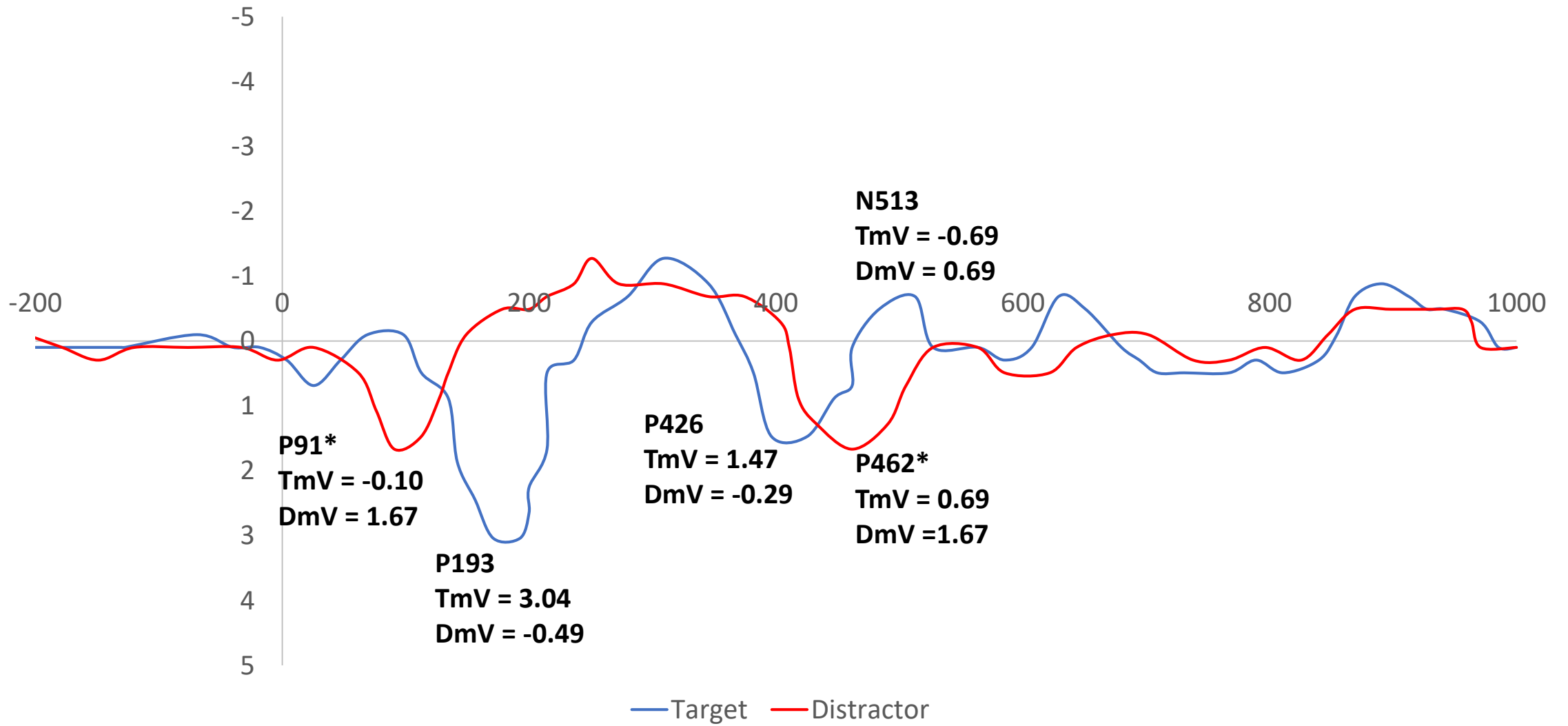

OZ

**N476**

**TmV = -3.42**

**DmV = -0.97**

**N598**

**TmV = -3.12**

**DmV = -1.03**

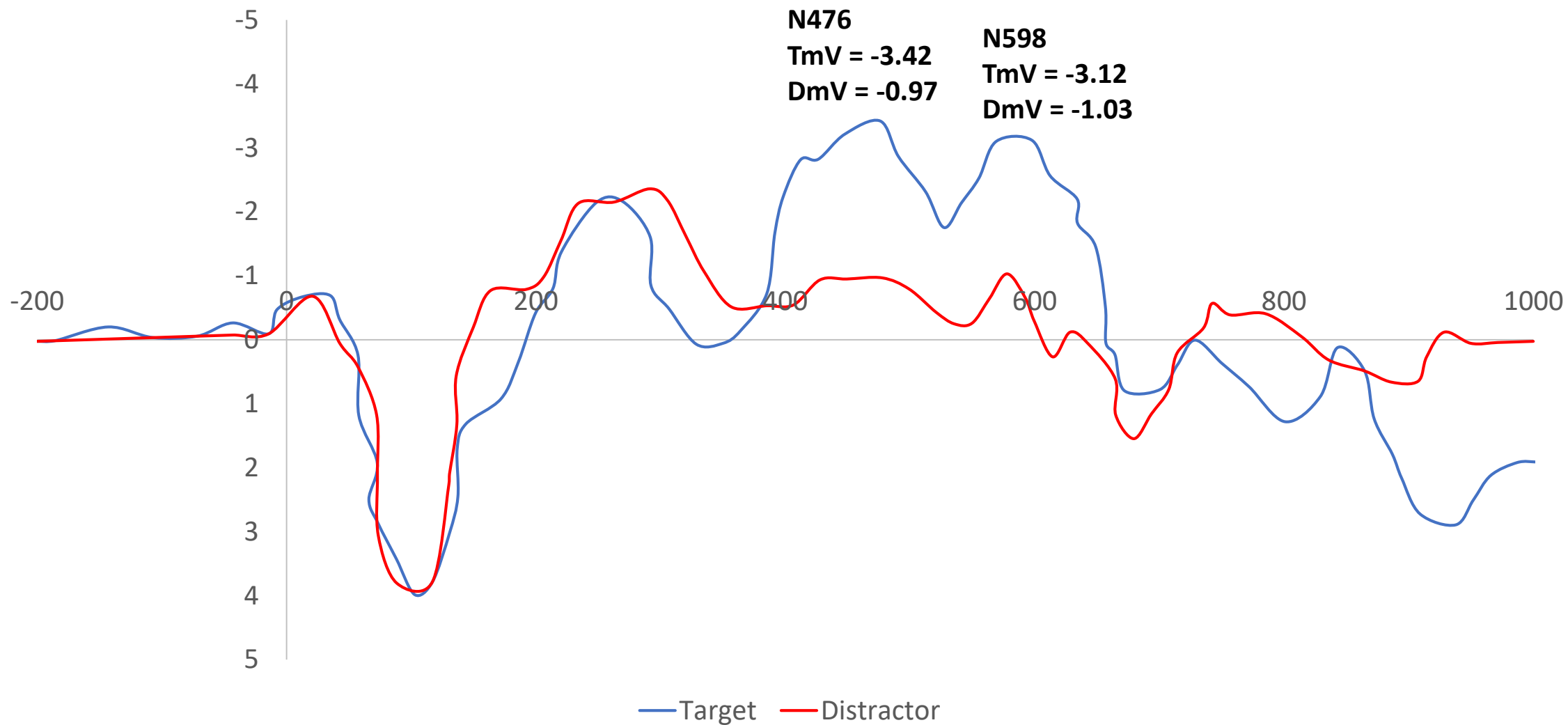

## O2

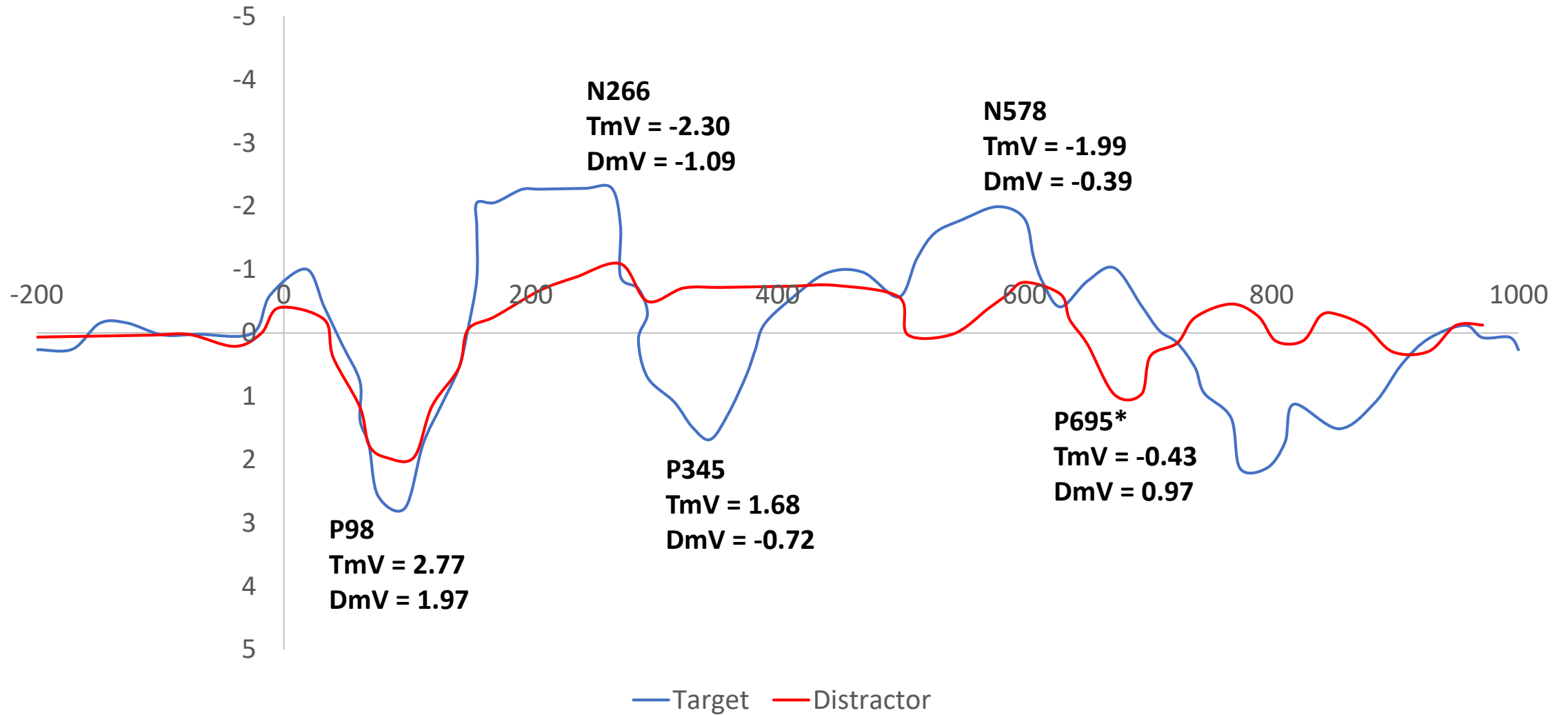

Supplement: Supplementary file 1 [file brainsci-10-00124-s001.pdf]
